# The cost evaluation of day-case compared to inpatient cochlear implantation in adults: subanalysis of a randomized controlled trial

**DOI:** 10.1007/s00405-024-08501-7

**Published:** 2024-03-26

**Authors:** Laura S. M. Derks, Adriana L. Smit, Hans G. X. M. Thomeer, Wilko Grolman, Robert J. Stokroos, Inge Wegner

**Affiliations:** 1 Department of Otorhinolaryngology-Head and Neck Surgery, G05.129, University Medical Center Utrecht, Heidelberglaan 100, 3584 CX Utrecht, The Netherlands; 2 Brain Center Rudolf Magnus, University Medical Center Utrecht, Utrecht, The Netherlands; 3 Jean Causse Ear Clinic, Traverse de Béziers, Colombiers, France; 4 Department of Otorhinolaryngology-Head and Neck Surgery, University Medical Center Groningen, Groningen, The Netherlands

**Keywords:** Cochlear implantation, Sensorineural hearing loss, Day-case, Inpatient, Cost evaluation

## Abstract

**Objective:**

To investigate the assumption that day-case cochlear implantation is associated with lower costs, compared to inpatient cochlear implantation, while maintaining equal quality of life (QoL) and hearing outcomes, for the Dutch healthcare setting.

**Study design:**

A single-center, non-blinded, randomized controlled trial in a tertiary referral center.

**Methods:**

Thirty adult patients with post-lingual bilateral sensorineural hearing loss eligible for unilateral cochlear implantation surgery were randomly assigned to either the day-case or inpatient treatment group (i.e., one night admission). We performed an intention-to-treat evaluation of the difference of the total health care-related costs, hospital and out of hospital costs, between day-case and inpatient cochlear implantation, from a hospital and patient perspective over the course of one year. Audiometric outcomes, assessed using CVC scores, and QoL, assessed using the EQ-5D and HUI3 questionnaires, were taken into account.

**Results:**

There were two drop-outs. The total health care-related costs were €41,828 in the inpatient group (*n* = 14) and €42,710 in the day-case group (*n* = 14). The mean postoperative hospital stay was 1.2 days (mean costs of €1,069) in the inpatient group and 0.7 days (mean costs of €701) for the day-case group. There were no statistically significant differences in postoperative hospital and out of hospital costs. The QoL at 2 months and 1 year postoperative, measured by the EQ-5D index value and HUI3 showed no statistically significant difference. The EQ-5D VAS score measured at 1 year postoperatively was statistically significantly higher in the inpatient group (84/100) than in the day-case group (65/100). There were no differences in postoperative complications, objective hearing outcomes, and number of postoperative hospital and out of hospital visits.

**Conclusion:**

A day-case approach to cochlear implant surgery does not result in a statistically significant reduction of health care-related costs compared to an inpatient approach and does not affect the surgical outcome (complications and objective hearing measurements), QoL, and postoperative course (number of postoperative hospital and out of hospital visits).

**Level of evidence:**

1.

**Supplementary Information:**

The online version contains supplementary material available at 10.1007/s00405-024-08501-7.

## Introduction

In the Netherlands, more than 500 patients undergo cochlear implant (CI) surgery for severe-to-profound sensorineural hearing loss (SNHL) each year [1]. A lot has changed, since the first cochlear implantation was performed in the Netherlands (Utrecht) in 1985 [2]. Where at the time patients were admitted to the ward for multiple days, it is now progressively being performed as a day-case procedure. Refinement of the surgical technique, improvement of the anesthetics, and the overall strive toward minimalizing hospital stay [3] contribute to the feasibility of this day-case approach. Another major drive toward day-case surgery is the potential financial benefit of a shorter hospital stay.

In the Netherlands, adult patients eligible for cochlear implantation receive reimbursement for only one CI, since bilateral implantation is reserved for patients up to 18 years old. The Dutch health insurance companies pay a pre-negotiated price for the full treatment of a diagnosis. In CI surgery, this full treatment encompasses the outpatient visits, imaging, surgery and hospitalization, and the (paramedic) aftercare. The average costs of hospitalization as indexed in 2015 are approximately 650 euros, costs that can be reduced when performing the procedure in a day-case setting [4]. However, to justify this potential cost-saving, this should not negatively affect the safety and hearing related benefit of cochlear implantation procedures between a day-case and inpatient approach. In a previous study, we demonstrated that there were no clinically relevant postoperative differences in quality of life (QoL), patient satisfaction, objective and subjective hearing outcomes, and postoperative complications between day-case and inpatient cochlear implantation [5]. In addition, the aim of the subanalysis of this randomized controlled trial is to assess the difference in mean health care-related costs between day-case and inpatient cochlear implantation.

## Methods

This article is based on data acquired in a single-center, non-blinded, randomized controlled trial, approved by the Institutional Review Board of the University Medical Center Utrecht (NL45590.041.13). This study was registered in the Netherlands Trial Register (http://www.trialregister.nl; NTR4464, March 13th 2014). The complete study protocol was published in October 2016 [6]. Due to currents insights concerning cost analyses, we decided to perform a descriptive cost evaluation in contrary to what was initially described in our study protocol.

### Study population

**Fig. 1:**
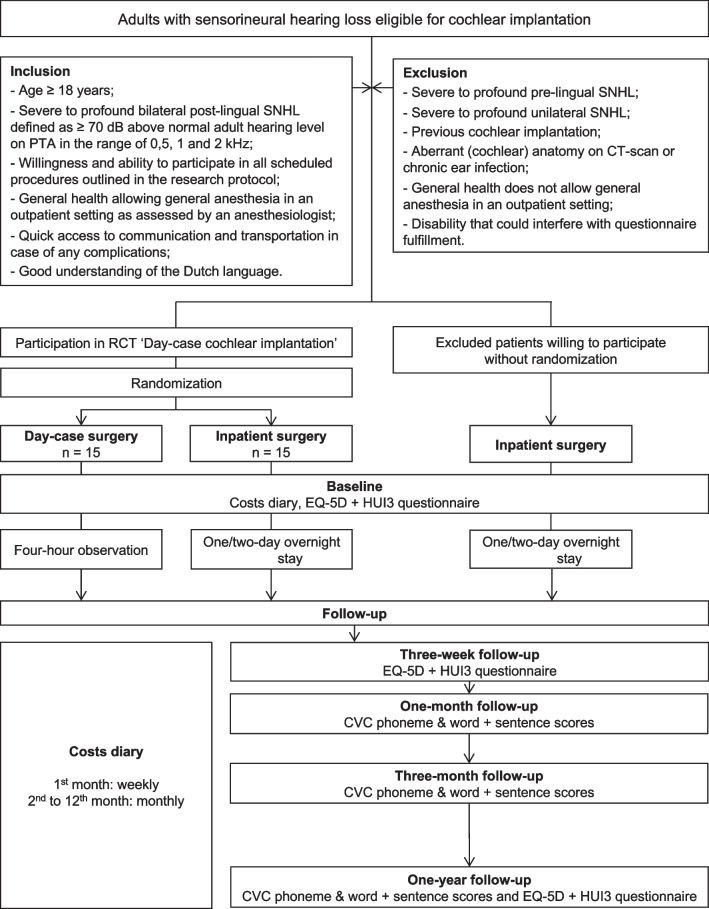
Flowchart of day-case cochlear implantation study. *SNHL* sensorineural hearing loss, *dB* decibel, *PTA* pure-tone audiometry, *n* number of patients, *EQ*-*5D* EuroQol-5D, *HUI3* Health Utilities Index-Mark 3, *CVC* consonant–vowel-consonant

Patients undergoing a unilateral cochlear implantation were eligible to participate if they met all of the inclusion criteria shown in Fig. 1.

### Sample size calculation

A sample size calculation was performed based on the primary outcome measure of the trial: general QoL on the Health Utilities Index-Mark 3 (HUI3) [6]. Fourteen participants per group were needed. To anticipate 10% withdrawal of participants, a total of 30 participants were recruited.

### Randomization, blinding, and treatment allocation

Patients were enrolled by one of two researchers (authors LD and IW). After inclusion, patients were allocated to either the inpatient group or the day-case group using a web-based randomization program (Julius Center, UMC Utrecht, Utrecht, The Netherlands). Patients were randomly allocated into two groups with stratification for age. Block randomization was used with an allocation ratio 1:1. The randomization chart, including block size, was established by an independent data manager before the start of the study. Consequently, treatment allocation sequence was concealed for patients, care providers, and researchers. Blinding of the involved was not possible, because patients and care providers would be aware of the surgical setting and hospital stay. Crossover was defined as admission of a day-case patient or discharge on the day of surgery of patients allocated to the inpatient group. Crossover between groups and causes thereof were assessed using patients’ charts. Readmission was defined as admission after initial discharge. In case of crossover, patients were asked to complete their follow-up, and analyses were carried out on an intention-to-treat basis.

### Baseline

Baseline characteristics were assessed using patients’ charts. Preoperatively, patients were asked to fulfill a costs diary (Appendix 1) and the EuroQol-5D (EQ-5D) [7–9] and Health Utilities Index-Mark 3 (HUI3) [10, 11] questionnaires as a baseline measurement.

### Intervention

The cochlear implant placements were performed under general anesthesia by one of three surgeons (authors AS, HT, and VT) in the same tertiary referral center (University Medical Center Utrecht). Patients allocated to the inpatient group were admitted one day before or on the day of surgery and were discharged 1–2 days after surgery. Patients allocated to the day-case group were admitted 1 day before or on the day of surgery and were discharged the day of surgery. If postoperatively patients were judged by the surgeon as not being physically capable of same-day discharge (i.e., due to postoperative nausea or dizziness) or if the surgeon did not support same-day discharge based on specific reasons, this was noted and patients would stay overnight.

### Cost evaluation

The cost evaluation was performed from a hospital and patient perspective and included hospital and out of hospital costs, starting from surgery to 1 year postoperatively. Hospital costs included outpatient clinic visits, diagnostic tests, surgery, hospitalization, and postoperative complications, and were assessed using patients’ charts. Out-of-hospital costs included (unscheduled) general practitioner visits and productivity-related costs which were documented using costs diaries. These were fulfilled weekly during the first month after surgery and monthly for the rest of 1 year.

For both groups the mean costs per patient were calculated using the number of used resources, multiplied by the unit price. Because of the confidentiality of these unit prices, an overall total cost per patient and total postoperative admission cost were given. For all other in and out of hospital cost items, the number of visits or number of tests was given. The unit prices of out of hospital costs (i.e., general practitioner visits and productivity-related costs) were calculated using the Dutch guidelines for costing research in health economic evaluations, issued by the National Healthcare Institute [4]. Published data of cumulative complications in large cohorts were used to determine weighted costs of complications [12].

The unit prices were corrected to June 2023 with an inflation price index number [13]. All costs were expressed in 2023 Euros (Exchange rate of September 23, 2023: €1 = US $1.07/ = £0.87).

### Audiometric evaluation

Audiometric evaluation consisted of repeating a set of Dutch words with a consonant–vowel-consonant structure (CVC) and Dutch sentences in a free field setting. The outcome measures were the percentage of correctly repeated words, phonemes, and complete sentences. The measurements were performed under optimal conditions using the cochlear implant and with a hearing aid in the contralateral ear if one was used in daily life situations. Audiometric evaluation was performed at approximately 1 month, 3 months, and 1 year postoperatively.

### Quality of life

The QoL was assessed using the EQ-5D and HUI3 questionnaires which were fulfilled at 3 weeks and 1 year postoperatively. The EQ-5D questionnaire is a five-item questionnaire on mobility, self-care, daily activities, pain and complaints, and anxiety or depression and a visual analog scale (VAS). Each domain is scored from 1 (no problems) to 3 (extreme problems), resulting in a single index value of the general health status between − 0.33 and 1. With the VAS, the general health status is rated from 0 (worst imaginable health status) to 100 (best imaginable health state) [7–9]. The HUI3 is a 15-item questionnaire that measures general health by evaluating eight domains: vision, hearing, speech, ambulation, dexterity, cognition, emotion, and pain. The outcome is a multi-attribute health status resulting in a utility score between -0.36 (state worse than death) and 1.00 (perfect health) [10, 11].

### Statistical analyses

Means and SDs were calculated for all baseline characteristics per group. Differences in the baseline were analyzed using the Mann–Whitney *U* test for continuous variables and the Fisher’s exact test for categorical variables. Normality was analyzed using means, medians, histograms, and box-plots. Non-parametric tests were used as none of the outcome data were normally distributed. A *p* value < 0.05 (two-tailed) was considered statistically significant. The mean health care-related costs difference between the day-case and inpatient group was calculated. For the other outcome data, means and SDs were calculated. Between-group mean differences with 95% confidence intervals (CIs) were calculated. The Mann–Whitney *U* test was used for further analyses of between-group differences.

Missing values were handled using multiple imputation. We assumed that the missing data were missing at random. Ten imputed datasets were created based on the correlation between variables with missing data and other variables. Pooled data of these ten datasets were used for further analyses.

All analyses were performed on an intention-to-treat basis and were performed using SPSS version 25.0 (SPSS Inc., Chicago, IL).

## Results

### Patient flow

**Fig. 2:**
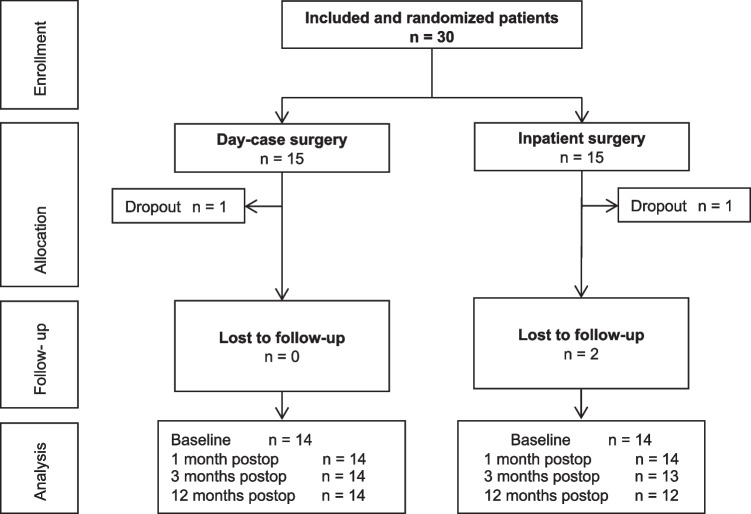
Flowchart. *n* number of patients

A total of 30 patients were included from April 2014 to April 2019 (Fig. 2). Follow-up took place from April 2014 to July 2020. A total of 28 (93%) cases received treatment. Two patients dropped out after treatment allocation and before receiving treatment: one in each randomization group. In one patient, the surgery was canceled because of severe cardiologic comorbidity and one patient requested cancelation of the operation. The two drop-outs were excluded from all analyses.

There were no missing data regarding the hospital costs and missing data in the out of hospital costs were handled using multiple imputation.

### Baseline characteristics

There were no significant between-group differences in the baseline characteristics (Table 1).

**Table 1:** Baseline characteristics

	Inpatient (*n* = 14)	Day-case (*n* = 14)	Mean difference (95% CI)
Sex (male:female)	5:9	6:8	
Age at surgery (mean (SD) in years)	59 (13)	62 (13)	− 2*
Cause of deafness [*n* (%)], operated side			
Congenital SNHL	2 (14)		
Hereditary hearing loss	4 (28)	2 (14)	
Sudden deafness	2 (14)	1 (7)	
Meniere’s disease		3 (21)	
Otosclerosis	3 (21)		
Meningitis		2 (14)	
Toxoplasmosis		1 (7)	
Progressive hearing loss	1 (7)	3 (21)	
Other	1 (7)	2 (14)	
Unknown	1 (7)		
Cost diary			
Highest completed educational training [*n* (%)]	2 (14)	2 (14)	
Primary school	3 (21)		
Preparatory/lower vocational education	2 (14)	2 (14)	
Intermediate secondary education	1 (7)	1 (7)	
Intermediate vocational education		2 (14)	
Higher vocational/pre-university education	1 (7)	3 (21)	
University of Professional Education	2 (14)	1 (7)	
College	1 (7)	2 (14)	
Other	2 (7)	1 (7)	
Missing			
Occupation in everyday life [*n* (%)]			
Work in paid employment	6 (43)	4 (29)	
Self-employed	1 (7)	2 (14)	
Housewife/-husband	3 (21)	1 (7)	
Unemployed		1 (7)	
Unfit for work	1 (7)	1 (7)	
Retired	2 (14)	5 (36)	
Other	1 (7)		
Paid employment; yes [*n* (%)]	8 (57)	6 (43)	
Hours of work per week (mean (SD) in hours)	34 (14)	36 (10)	− 2 (− 17 to 13)*
Days at work per week (mean (SD) in days)	4 (1)	5 (1)	1 (− 2 to 1)*
Perioperative characteristics			
Side of surgery (left:right)	4:10	3:11	
Implant, brand [*n* (%)]			
Cochlear	10 (71)	7 (50)	
MED-EL		3 (21)	
Advanced bionics	4 (29)	3 (21)	
Oticon		1 (7)	

*n* number of patients, *SD* standard deviation, *SNHL* sensorineural hearing loss

*Independent-samples Mann–Whitney *U* test

Fisher’s exact test (2-sided). None of the baseline characteristics were statistically significant

### Cost evaluation

**Fig. 3:**
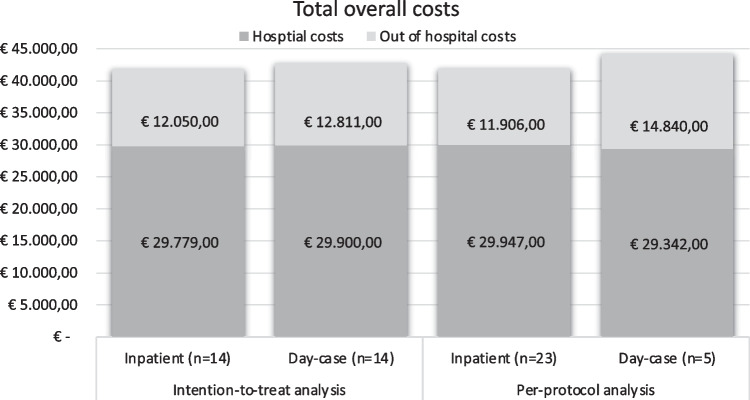
Total health care-related costs. *n* number of patients

The total health care-related costs were €41,828 (SD 8198) in the inpatient group and €42,710 (SD 11,934) in the day-case group with a statistically non-significant mean difference of − €882 (95% CI − 8836 to 7072). In the inpatient group, the total hospital costs were €29,779 (SD 925) and the out of hospital costs €12,050 (SD 8342) and in the day-case group €29,900 (SD 1594) and €12,811 (SD 11,967), respectively (Tables 2, 3; Fig. 3).

**Table 2:** Evaluation of hospital costs

	Inpatient (*n* = 14)	Day-case (*n* = 14)	Mean difference (95% CI)
Perioperative hospital costs and stay			
Costs (mean (SD) in euros)			
Total hospital costs	29,779 (925)	29,900 (1594)	− 121 (− 1133 to 892)*
Postoperative hospital stay	1,069 (375)	701 (449)	367 (46 to 688)*
Hospitalization (mean days (SD))			
Duration postoperative stay	1.2 (0.4)	0.7 (0.6)	0.5 (0.1 to 0.9)*
Duration surgery (mean minutes (SD))	121 (27)	125 (30)	− 4 (− 26 to 18)*
Postoperative hospital visits			
Outpatient clinic visits (mean number (SD))			
Otolaryngologist	1.9 (0.8)	2.9 (2.4)	− 1 (− 2.4 to − 0.4)*
Outpatient clinic consultation	0.4 (0.8)	0.1 (0.3)	0.3 (− 0.2 to 0.8)*
Telephone consultation			
Audiologist	14 (2.1)	15.3 (5.3)	− 1.3 (− 4.4 to 1.9)*
Outpatient clinic consultation	0.4 (0.6)	0.7 (0.3)	0.3 (− 0.1 to 0.7)*
Telephone consultation	1 (0.4)	1.2 (0.6)	− 0.2 (− 0.6 to 0.2)*
Social worker and psychologist	0.4 (0.7)	0.6 (1.2)	− 0.2 (− 1.0 to 0.6)*
Other			
Emergency department visits	0 (0)	0 (0)	
Tests (mean number (SD))			
Audiometric evaluation	1.3 (0.6)	1.5 (1.6)	− 2 (− 1.1 to 0.7)*
ENG	0.1 (0.3)	0 (0)	0.1 (− 0.1 to 0.2)*
CT-scan	0.1 (0.3)	0.1 (0.3)	0 (− 0.2 to 0.2)*

*CI* confidence interval, *CT* computed tomography, *ENG* electronystagmography, *n* number of patients, *SD* standard deviation

*Independent-samples Mann–Whitney *U* test; differences printed in bold were statistically significant (*p* < 0.05). All costs are expressed in 2022 euros

**Table 3:** Evaluation of out of hospital costs

	Inpatient (*n* = 14)	Day-case (*n* = 14)	Mean difference (95% CI)
Total out of hospital costs [mean (SD) in euros]	12,050 (8342)	12,811 (11,967)	− 762 (− 8775 to 7252)*
Sick leave (mean number of days (SD))			
Paid work			
First month postoperative	7.6 (9.7)	6.0 (12.8)	1.6 (− 6.7 to 10.0)*
Total year	10.8 (11.7)	10.1 (20.5)	0.7 (− 11.7 to 13.0)*
Unpaid work (voluntary work/housework)			
First month postoperative	13.9 (6.9)	14.9 (9.0)	
Total year	44.6 (12.7)	52.2 (26.7)	− 1.0 (− 7.0 to 4.9)*
Psychological or physical problems on workdays (mean number of days (SD))^$^			− 7.6 (− 22.5 to 7.4)*
First month postoperative	0.3 (0.8)	1.1 (2.1)	
Total year	2.9 (4.1)	4.4 (8.0)	− 0.8 (− 2.0 to 0.3)*
Psychological or physical problem VAS score (mean (SD))			− 1.5 (− 6.2 to 3.1)*
First month postoperative	0.2 (0.4)	0.8 (1.6)	
Total year	0.4 (0.6)	0.6 (0.9)	
Extra help (hours (SD))			− 0.6 (− 1.4 to 0.3)*
First month postoperative	10.8 (6.1)	15.3 (9.1)	− 0.2 (− 0.8 to 0.3)*
Total year	30.9 (11.0)	37.8 (15.7)	− 4.5 (− 10.4 to 1.4)*
Consultation primary care physician during working hours (number of visits (SD))			− 7.0 (− 16.9 to 3.0)*
First month postoperative	1.2 (0.9)	1.5 (1.0)	
Total year	5.1 (3.2)	6.7 (4.0)	− 0.3 (− 0.9 to -0.4)*
Consultation primary care physician outside working hours (number of visits (SD))			− 1.7 (− 4.3 to 1.0)*
First month postoperative	0 (0)	0 (0)	
Total year	0.3 (0.5)	0.3 (0.5)	
Consultation speech therapist (number of visits (SD))	0.4 (0.4)	0.4 (0.4)	− 0.0 (− 0.4 to 0.3)*
First month postoperative	1.0 (0.7)	0.9 (0.3)	0.0 (− 0.2 to 0.2)*
Total year			0.1 (− 0.3 to 0.6)*
Consultation physiotherapist (number of visits (SD))	0.2 (0.3)	0.3 (0.4)	
First month postoperative	1.0 (0.8)	1.1 (0.9)	− 0.0 (− 0.3 to 0.3)*
Total year			− 0.1 (− 0.7 to 0.6)*

*CI* confidence interval, *n* = number of patients, *SD* standard deviation

*Independent-samples Mann–Whitney *U* test; none of the out of hospital costs were statistically significant

The mean postoperative hospital stay for the inpatient group was 1.2 days (0.4) with a mean cost of €1069 (SD 375) and for the day-case group 0.7 days (SD 0.6) with a mean cost of €701 (SD 449). The mean difference in number of days [0.5 (95% CI 0.1–0.9)] was statistically significant, while the mean difference in costs for hospital stay (€367 (95% CI 46–688)) was not statistically significant. There were no significant differences in the number of postoperative hospital visits or postoperative diagnostics tests (audiometric evaluation, ENG or CT-scan). None of the patients visited the emergency department in the first postoperative year (Table 2).

In four day-case patients, postoperative complications resulted in a prolonged hospital admission: postoperative nausea and vomiting (*n* = 1), at request of the surgeon for longer observation time after intraoperative complications [gusher (without postoperative vertigo) *n* = 1, hypertension *n* = 1, type IV allergic reaction to dexamethasone *n* = 1].

Other reasons for crossover of the day-case patient were drowsiness (*n* = 1), late scheduled surgery (*n* = 2), social reasons (*n* = 1), or due to an unclear reason (*n* = 1). A total of 9 out of 14 patients (64%) allocated to the day-case group were admitted to the ward after surgery for one (*n* = 8) or two (*n* = 1) nights. Early postoperative complications were vertigo (*n* = 5 inpatient; *n* = 3 day-case) and wound infection (*n* = 1 day-case), resulting in extra outpatient clinic visits.

The out of hospital costs are shown in Table 3. None of the differences between the inpatient and day-case group were statistically significant.

### Audiometric evaluation

The mean CVC phoneme score did not differ significantly between the inpatient and day-case group at all three follow-up moments. The mean CVC word scores were statistically significantly lower at 1 month postoperatively in the day-case group (69% versus 57%) and were not statistically significantly different at 3 months and 1 year postoperatively. The mean sentence test scores were not significantly different between both groups (Table 4).

**Table 4:** Hearing evaluation outcomes

	Inpatient (*n* = 14)	Day-case (*n* = 14)	Mean difference (95% CI)
CVC phoneme score (mean % (SD))^#^			
1 month postop	85 (13)	74 (21)	10 (− 3 to 23)*
3 months postop	88 (13)	79 (22)	9 (− 4 to 22)*
1 year postop	89 (9)	81 (20)	8 (− 3 to 19)*
CVC word score (mean % (SD))^#^			
1 month postop	69 (19)	57 (25)	**13 (**− 4 to 29)*
3 months postop	76 (20)	63 (30)	12 (− 6 to 31)*
1 year postop	78 (16)	66 (28)	12 (− 4 to 28)*
Sentence test score (mean % (SD))^¥^			
1 month postop	87 (23)	80 (28)	7 (− 12 to 26)*
3 months postop	90 (15)	92 (13)	− 1 (− 12 to 9)*
1 year postop	90 (16)	90 (21)	1 (− 12 to 14)*

*CI* confidence interval, *CVC* consonant–vowel-consonant, *SD* standard deviation

^#^CVC phoneme and word score at 65 dB SPL in quiet

^¥^Sentence test score: percentage of correctly replied sentences

*Independent-samples Mann–Whitney *U* test. Differences printed in bold were statistically significant (*p* < 0.05)

### Quality of life

The postoperative EQ-5D index value was 0.97 and 0.91 in the inpatient group and 0.89 and 0.72 in the day-case group at 3 weeks and 1 year, respectively. Both mean differences (0.07 and 0.19) were not statistically significant. The EQ-5D VAS score was 80 in the inpatient group and 73 in the day-case group at three weeks postoperatively with a mean difference of 7 that was not statistically significant. At 1 year postoperatively, the EQ-5D VAS scores were 84 in the inpatient group and 65 in the day-case group with a statistically significant mean difference of 19.

The overall QoL, measured with the HUI3, at 3 weeks postoperatively was equal in both groups (mean difference = 0.09 points) (Table 5). The total score differed 0.17 at 1 year postoperatively between groups (not statistically significant).

**Table 5:** Outcomes of Quality-of-Life questionnaires

	Inpatient			Day-case			Difference (95% CI)		
	Preoperative	3 weeks postop	1 year postop	Preoperative	3 weeks postop	1 year postop	Preoperative	3 weeks postop	1 year postop
EQ-5D (mean (SD)) Index value	*n* = 14 0.91 (0.11)	*n* = 14 0.97 (0.67)	*n* = 11 0.91 (0.13)	*n* = 14 0.87 (0.10)	*n* = 11 0.89 (0.13)	*n* = 11 0.72 (0.29)	0.02 (− 0.01 to 0.10)*	0.07 (− 0.02 to 0.15)*	0.19 (− 0.01 to 0.39)*
EQ-5D (mean (SD)) VAS score	*n* = 14 75 (22)	*n* = 14 80 (13)	*n* = 11 84 (7)	*n* = 14 79 (9)	*n* = 11 73 (11)	*n* = 10 65 (25)	− 3 (− 16 to 10)*	7 (− 3 to 17)*	19 (3 to 35)*
HUI3 (mean (SD)) Total score	*n* = 14 0.52 (0.20)	*n* = 14 0.58 (0.19)	*n* = 11 0.68 (0.18)	*n* = 14 0.50 (0.13)	*n* = 11 0.49 (0.16)	*n* = 11 0.51 (0.27)	0.02 (− 0.10 to 0.14)*	0.09 (− 0.05 to 0.23)*	0.17 (− 0.02 to 0.37)*

*EQ-5D* EuroQol-5D, *VAS* visual analog scale, *HUI3* Health Utility Index 3, *n* number of patients, *SD* standard deviation, *CI* confidence interval

*Independent-samples Mann–Whitney *U* test

^^^Fisher’s exact test. Differences printed in bold were statistically significant (*p* < 0.05)

## Discussion

This study allows for an evaluation of the health care-related hospital and out of hospital costs (hospital and patient perspective), between day-case and inpatient cochlear implantation in adult taking the hearing outcome and QoL into account. With our intention-to-treat analysis, we found a difference of -€882 in the total health care-related costs of cochlear implantation and the 1-year postoperative care between both groups that was not statistically significant (mean inpatient = €41,828; mean day-case = €42,710). There were no statistically significant differences in the number of postoperative outpatient visits or diagnostics tests.

Due to the relatively large crossover rate (64%; 9/14 patient) from the day-case to inpatient group, we performed a per-protocol analysis to compare the true difference between a day-case an inpatient approach showing mean total health care-related costs of €41,854 (SD 9257) in the inpatient group (*n* = 23) and €44,182 (SD 14,344) in the day-case group (*n* = 5) with a mean difference of − €2329 (95% CI − 12,681 to 8023) that was not statically significant (Fig. 3). The total postoperative hospital days for the inpatient group were 1.2 (SD 0.4) with a mean total cost of €1033 (SD 340) compared to a mean total cost of day-case of €203 (SD 0), showing a statistically non-significant mean difference of €829 (95% CI 511–1147). With approximately 500 cochlear implant surgeries per year [1], this means a potential cost reduction of hospital admission of €414,500 (500 × €829) per year in the Netherlands.

What is most important is that the cost reduction does not go at the expense of the QoL or hearing outcomes of patients. In this study, we found no between-group differences in the postoperative objective hearing outcome (equal CVC scores) or the QoL (equal EQ-5D index value and HUI3). We did find a statistically significant difference in EQ-5D VAS score of 19 (84 in the inpatient group and 65 in the day-case group) at 1 year postoperatively, meaning that the day-case group rated their general health status 19 out of 100 points worse 1 year postoperatively than the inpatient group. Preoperatively, the VAS scores were almost equal for both groups (75 in the inpatient group and 79 in the day-case group) and a minimal difference of 7 points was found at 3 weeks postoperatively that was not statistically significant (80 inpatient group vs. 73 day-case group). It is unlikely that the allocation to the day-case group is the cause of the relatively large difference in general health status at 1 year postoperatively.

Unfortunately, there is only one other study comparing the costs between day-case and inpatient cochlear surgery. Teschner et al. [14] compared cochlear implant surgery between a clinic in Germany (4-day hospital stay postoperatively) and a clinic in the United States (day-case). They found an average reimbursement of €30,802 in Germany and €19,643 in the United States, resulting in a €11,159 difference in reimbursement between day-case and a 4-day hospital stay. However, the different healthcare systems, especially that of the United States makes an actual comparison with our outcome questionable.

When evaluating the potential cost reduction of a day-case approach in other surgical fields, our results are comparable to those described in the literature. One article evaluated the cost difference between day-case and inpatient elective percutaneous coronary intervention in the Netherlands [15]. They found a mean difference of €258 in favor of the day-case approach, mainly due to the overnight stay in the inpatient group as all other costs were equal for both treatment groups. Borakati et al. [16] found a cost reduction of £529 (€611) when performing arthroplasty in a day-case setting in a general hospital in London. Ignat et al. [17] assessed performing bariatric surgery in a day-case setting (University Hospital in Strasbourg, France) and found a 14% cost reduction (€721) compared to an inpatient group.

In interpreting our findings, the following considerations need to be taken into account. Due to the large crossover rate of 64%, the outcome of the intention-to-treat analysis may not be representative for the difference in costs between a day-case and inpatient surgical approach. This may limit the generalizability of outcomes. Also, the sample size calculation was based on the primary outcome measure of the original randomized controlled trial: general QoL on the Health Utilities Index-Mark 3 (HUI3). This study, with a sample size of 28, may not be sufficient to detect a significant difference in costs between both groups. Another limitation is that with the use of questionnaires (EQ-5D and HUI3) and cost diaries, there is a risk of self-reporting bias and recall bias. As this would have influenced outcomes of both groups, we expect that this will not largely influence the overall conclusions. Also, within the questionnaires and cost diaries these was a varying degree of missing data. Although handled using multiple imputations, ideally analyses would have been performed without missing data. Finally, different health care systems with differences in financial coverage of medical procedures across the world make it difficult to compare our results to those of other countries.

## Conclusion

Overall, a day-case approach to CI surgery does not result in a statistically significant reduction of health care-related hospital and out of hospital costs compared to an inpatient approach and does not affect the surgical outcome (complications and objective hearing measurements), QoL, and postoperative course (number of postoperative hospital and out of hospital visits). However, with the increasing shortages of hospital staff and beds performing cochlear implantation in a day-case setting seems a safe and effective approach which can facilitate limiting the use of health care resources for cochlear implantation.

### Supplementary Information

Below is the link to the electronic supplementary material.

Supplementary file1 (PDF 160 KB)
