# Supplementary material for: The cost evaluation of day-case compared to inpatient cochlear implantation in adults: subanalysis of a randomized controlled trial
Source: Eur Arch Otorhinolaryngol. 2024 Mar 26;281(8):4009–19. doi: 10.1007/s00405-024-08501-7 (PMC11266201; doi:10.1007/s00405-024-08501-7)
Supplement: Supplementary file 1 — Supplementary file1 (PDF 160 KB) [file 405_2024_8501_MOESM1_ESM.pdf]

UMC Utrecht

Costs diary

This costs diary regards **week / month** * ____________________ of the year _________

Date: ____ / ____ / _______

Unique participation number: ____

Treatment group: **day-case surgery / inpatient surgery** *

*** Delete as applicable**

Question 1 and 2 will be filled in once, only at intake:

1. **What is your highest <u>completed</u> educational training?**

0 No school or training completed

0 Primary school

0 Preparatory vocational education / lower vocational education

0 Intermediate secondary education

0 Intermediate vocational education

0 Higher vocational education / pre-university education

0 University of Professional Education (UPE)

0 College

0 Other: ________________________

2. What do you do in everyday life?

0 I am in school/college

0 I work in paid employment

0 I am self-employed

0 I am housewife, -husband

0 I am unemployed

0 I am unfit for work

0 I am retired

0 Other: ________________________

<u>Part A. Questions regarding work</u>

3. Do you have a paid employment?

0 No. *Proceed to question 13.*

0 Yes, ik heb betaald werk. *Proceed to question 4.*

4. What is your profession?

____

5. How many hours a week do you work?

Only count the hours you are being paid for.

_______ hours

6. On how many days a week do you work?

_______ days

7. Were your absent at work in the past 4 weeks <u>due to illness?</u>

0 No

0 Yes, I have been absent for ________________ *<u>work</u>days*

8. Were you absent at work longer that the duration of 4 weeks due to illness?

This concerns a continuous period of absence.

0 No

0 Yes

9. What date did you call in sick?

Date: ___ / ___ / ______

UMC Utrecht

10. Were there days in the past 4 weeks on which you did attend work, but during which you suffered from psychiatric or physical problems during work?

0 No

0 Yes

11. On how many workdays did you suffer from psychiatric or physical problems during work?

Only count the workdays in the past 4 weeks __________ workdays

12. On the days that you suffered from these problems, it is possible that you performed your work less effective than usual. Can you give an indication of this on the scale below?

Look at the numbers below. Number 10 indicates that on these days you were able to perform work as effective as usual. Number 0 indicates that you could nog perform your work at all on these days. Encircle the applicable number.

I could not perform work on these days				I could perform approximately half of work					I could perform work as effective as usual	
0	1	2	3	4	5	6	7	8	9	10

.

Also in unpaid work (for example: voluntary work, the housework, work in the garden, doing groceries) it is possible to suffer from psychiatric or physical problems

13. Were there days in the past 4 weeks on which you could perform less unpaid work due to suffering from psychiatric or physical problems?

0 No

0 Yes

14. On how many days was this the case? _______ days

15. Suppose that someone, for example your partner, relative or an acquaintance, would have helped you on these days. And would have performed the unpaid work that you were not able to do for you. How many hours would that person have been busy with this on average on these days?

___________ hours

Part B. Questions regarding care

16. What medication have you used in the past 4 weeks?

0 No medication

0 Medicine 1: name: ____

0 Medicine 2: name: ____

0 Medicine 3: name: ____

0 Medicine 4: name: ____

0 Medicine 5: name: ____

0 Medicine 6: name: ____

0 Medicine 7: name: ____

0 Medicine 8: name: ____

17. How many appointments have you had with your family doctor in the past 4 weeks?

0 No appointments

0 ___________ appointments *during daytime on workdays*

0 ___________ appointments *on workdays outside working hours or in the weekend*

18. Did you have an appointment at the outpatient clinic of the hospital in the past 4 weeks?

This concerns appointments with a doctor for yourself. For example: cardiologist, rheumatologist, ENT specialist, neurologist.

0 No

0 Yes

19. Which doctors have you visited in the past 4 weeks? And how often?

	Doctor:	Number of times:
For example:	*Cardiologist*	*2 times*
1	______________	______ times
2	______________	______ times
3	______________	______ times
4	______________	______ times
5	______________	______ times
6	______________	______ times

20. Did you have an appointment with one or more of the caregivers mentioned below in the past 4 weeks? If so, how often?

	Caregiver:	Number of times:
0	Physiotherapist	______ times
0	Occupational therapist	______ times
0	Speech therapist	______ times
0	Dietician	______ times
0	Social worker	______ times
0	Company doctor	______ times
0	Audiologist	______ times
0	Psychologist / psychotherapist	______ times
0	Other, ______________	______ times

UMC Utrecht

21. How many times have you visited the Emergency Room (ER) in the hospital in the past 4 weeks?

0 I have nog visited the ER.

0 I have visited the ER _______ times.

22. Have you been admitted to the hospital in the past period?

During a hospital admission you sleep over in the hospital, for example if you are not allowed to leave the hospital after an operation.

A day-case admission is an admission whereby you do not sleep over in the hospital, for example with chemotherapy treatment, dialysis or blood transfusions. This also includes a day of rehabilitation in a rehabilitation centre.

If your were admitted more than once for either hospital or day-case admission, sum up the total number of days.

0 No

0 Yes, for hospital admission ____________ days

0 Yes, for day-case admission ____________ days

23. Have you made costs this week for required extra help?

0 No

0 Yes:
- 0 Childcare, approximately € _______________
- 0 Household, approximately € _______________
- 0 Overig, namelijk:
  - 0 (reason) _____________, approximately € _________
  - 0 (reason) _____________, approximately € _________
  - 0 (reason) _____________, approximately € _________

Thank you for completing this questionnaire!

You will receive notification when your next questionnaire is available.
